# 3D Bioprinting of Pectin-Cellulose Nanofibers Multicomponent Bioinks

**DOI:** 10.3389/fbioe.2021.732689

**Published:** 2021-12-03

**Authors:** Matteo Pitton, Andrea Fiorati, Silvia Buscemi, Lucio Melone, Silvia Farè, Nicola Contessi Negrini

**Affiliations:** ^1^ Department of Chemistry, Materials, and Chemical Engineering “G. Natta”, Politecnico di Milano, Milan, Italy; ^2^ INSTM, National Consortium of Materials Science and Technology, Local Unit Politecnico di Milano, Milan, Italy; ^3^ Centro di Ricerca per l’Energia, l’Ambiente e il Territorio (CREAT), Università Telematica eCampus, Novedrate, Italy

**Keywords:** pectin, cellulose nanofiber, hydrogel, bioprinting, multicomponent bioink, 3D printing

## Abstract

Pectin has found extensive interest in biomedical applications, including wound dressing, drug delivery, and cancer targeting. However, the low viscosity of pectin solutions hinders their applications in 3D bioprinting. Here, we developed multicomponent bioinks prepared by combining pectin with TEMPO-oxidized cellulose nanofibers (TOCNFs) to optimize the inks’ printability while ensuring stability of the printed hydrogels and simultaneously print viable cell-laden inks. First, we screened several combinations of pectin (1%, 1.5%, 2%, and 2.5% w/v) and TOCNFs (0%, 0.5%, 1%, and 1.5% w/v) by testing their rheological properties and printability. Addition of TOCNFs allowed increasing the inks’ viscosity while maintaining shear thinning rheological response, and it allowed us to identify the optimal pectin concentration (2.5% w/v). We then selected the optimal TOCNFs concentration (1% w/v) by evaluating the viability of cells embedded in the ink and eventually optimized the writing speed to be used to print accurate 3D grid structures. Bioinks were prepared by embedding L929 fibroblast cells in the ink printed by optimized printing parameters. The printed scaffolds were stable in a physiological-like environment and characterized by an elastic modulus of E = 1.8 ± 0.2 kPa. Cells loaded in the ink and printed were viable (cell viability >80%) and their metabolic activity increased in time during the *in vitro* culture, showing the potential use of the developed bioinks for biofabrication and tissue engineering applications.

## Introduction

Three-dimensional (3D) bioprinting aims at combining cells and biomaterials to fabricate constructs layer-by-layer, with biological and physical properties recapitulating those of native tissues to be restored/replaced (Ashammakhi et al., 2019). Bioprinting of naturally derived polymer-based hydrogels represents a unique fabrication technology to obtain structures that provide a highly hydrated microenvironment, biocompatible, biodegradable, and able to promote and guide cell–cell and cell–extracellular matrix (ECM) interactions (Pereira and Bartolo, 2015; Ng et al., 2019). Despite the structure being reminiscent of the ECM, natural-derived polymers are generally characterized by batch-to-batch variability, uncontrolled degradation, and weak mechanical properties (Contessi Negrini et al., 2021). Printing naturally derived polymer-based hydrogels is consequently still challenging, as optimization of the mechanical properties and a suitable post-printing crosslinking strategy must be engineered to fabricate scaffolds with adequate shape fidelity (Mao et al., 2020). Several naturally derived polymers have been described as potential biomaterial inks/bioinks for different applications, including alginate, collagen, gelatin, and chitosan (Milazzo et al., 2019; Ng et al., 2019), but improvements in post-printing stability, accuracy, and shape fidelity of printed structures are still required (Ng et al., 2019). Among naturally derived polymers used to prepare biomedical hydrogels, pectin has recently attracted interest thanks to its high molecular weight, biocompatibility, easy availability, versatility, low cost, and its ability in mimicking the structure of polysaccharides found in the ECMs of mammals (Kumar et al., 2013; Mahendiran et al., 2021).

Pectin is a natural heteropolysaccharide extracted from plant cell walls by chemical or enzymatic processes (Koffi et al., 2013). Carboxyl groups of pectin can be esterified to form methyl esters. The degree of esterification DE (low-methoxylated pectin, DE < 50%, and high-methoxylated pectin, DE > 50%) is defined as the ratio of esterified carboxylic acid groups to the total number of carboxylic acids and it influences pectin gelling properties and solubility (Vancauwenberghe et al., 2017; Jacob et al., 2020). Pectin has been widely investigated for food and pharmaceutical applications (Munarin et al., 2012), and recently for biomedical applications, including cell (Mehrali et al., 2019) and drug delivery (Neufeld and Bianco-Peled, 2017; Zhu et al., 2019; Khotimchenko, 2020), wound dressing (Rezvanian et al., 2017; Oh et al., 2020), skin (Pereira et al., 2018a; Türkkan et al., 2018), and bone tissue engineering (Al-arjan et al., 2020; Markstedt et al., 2015; Nguyen et al., 2015; Zhao et al., 2016).

Pectin has been recently used for 3D printing applications, both as biomaterial ink (Banks et al., 2017; Vancauwenberghe et al., 2017; Vancauwenberghe et al., 2018; Vancauwenberghe et al., 2019; Stealey et al., 2019) and bioink (Pereira et al., 2018b; Vancauwenberghe et al., 2019). Advantages in the use of pectin for 3D printing are related to the shear-thinning behavior, since its viscosity decreases by increasing the applied shear rate, due to random coil rearrangement when a higher shear rate is applied, thus facilitating the extrusion process occurring during printing (Methacanon et al., 2014; Wang et al., 2014). However, pectin solutions generally exhibit relatively low viscosity values, especially at low concentrations (<1% w/v), hindering their printability (Methacanon et al., 2014; Cui et al., 2020). To improve pectin printability, its concentration has been increased to obtain shear-thinning inks (Methacanon et al., 2014). Alternatively, partial crosslinking *via* cations addition has been tested to obtain a suitable flow during the extrusion and a stable 3D structure after printing (Vancauwenberghe et al., 2017). However, these strategies might lead to a less permissive hydrogel microenvironment for cell encapsulation, and single-component pectin-based inks have been proved to be affected by printability limitations. Development of multicomponent pectin-based inks could represent an optimal alternative to improve pectin printability while maintaining its functionality (Cernencu et al., 2019; Cui et al., 2020). Multicomponent bioinks represent a unique approach for the biofabrication of functional and biomimetic tissue-like constructs (Ashammakhi et al., 2019), as they allow tuning the properties of the ink/bioinks to mimic human tissues complexity, by bioprinting multiple cell types and biomaterials while ensuring precise positioning of the deposed bioink layer-by-layer.

Here, we develop multicomponent bioinks based on pectin and TEMPO-oxidized cellulose nanofibers to improve pectin printability while ensuring a stable and biocompatible printed scaffold for potential use in tissue engineering. TEMPO-oxidized cellulose nanofibers were selected for their ability to crosslink in presence of polycations and nanoparticles (Fiorati et al., 2021), simultaneously improving the rheological properties of the prepared multicomponent bioinks. First, we prepared a set of multicomponent bioinks and selected the most promising ones by macroscopic evaluation of the printed structures. Then, we identified the most promising biomaterial inks and optimized the printing parameters to obtain multi-layered structures with good shape fidelity. Finally, we preliminary investigated *in vitro* the cytocompatibility of the developed bioinks and printing process to show the potential use of the developed materials in bioprinting-based tissue engineering applications.

## Materials and Methods

All materials were purchased from Sigma Aldrich unless differently specified. Pectin (PEC, from citrus peel, degree of esterification DE = 37%) was purchased from Herbstreith and Fox®. Pectin powder was sterilized by immersion in ethanol 70% v/v, followed by UV irradiation for 15 min. Cellulose nanofibers were sterilized by autoclaving. Culture medium: Dulbecco’s Modified Eagle Medium (DMEM), fetal bovine serum (FBS) 10% v/v, glutamine 2 mM, HEPES 10 mM, and penicillin/streptomycin 1x.

### Pectin-TOCNFs Multicomponent Inks Preparation

Multicomponent bioinks (P-C) were prepared by mixing pectin and 2,2,6,6-tetramethylpiperidine-1-oxyl radical (TEMPO)-Oxidized Cellulose Nanofibers (TOCNFs). TOCNFs were synthesized (Supplementary Figure S1) as previously described (Fiorati et al., 2020). TOCNFs dispersions were diluted 1:1 with DMEM (final concentrations = 0%, 0.5%, 1%, and 1.5% w/v). Then, pectin powder was gradually added (final concentrations = 1%, 1.5%, 2%, and 2.5% w/v), solutions were set for 4 h, CaCl_2_ 35 mM was added dropwise to partially pre-crosslink the solutions, and the ink pH was adjusted to 6–6.5 by addition of NaOH. Stirring was maintained until homogeneous pre-hydrogel inks were obtained. Pectin and TOCNFs were mixed to obtain a total of *n* = 16 ink formulations, by combining different pectin and TOCNFs concentrations (Table 1).

**TABLE 1:** Multicomponent pectin and TEMPO-Oxidized Cellulose Nanofibers (TOCNFs) hydrogels designed by varying the concentrations of the two components in the final inks. The first part of the name of the ink refers to the pectin concentration (Px), while the second part refers to the TOCNFs concentration (Cy).

	TOCNFs (% w/v)				
**Pectin (% w/v)**	—	0	0.5	1.0	1.5
	1.0	P1.0_C0	P1_C0.5	P1_C1	P1_C1.5
	1.5	P1.5_C0	P1.5_C0.5	P1.5_C1	P1.5_C1.5
	2.0	P2_C0	P2_C0.5	P2_C1	P2_C1.5
	2.5	P2.5_C0	P2.5_C0.5	P2.5_C1	P2.5_C1.5

### Characterization and Printing of Multicomponent Biomaterial Inks

The viscosity of the prepared inks was measured by rheological tests (Anton Paar MCR 302 Modular Compact rheometer, Anton Paar GmbH, Austria). Shear rate sweep tests (parallel plates, diameter Ø = 25 mm) were performed at 37°C by applying a shear rate ramp from 0.1 to 100 s^−1^ (Paxton et al., 2017).

Inks were printed using a customized robotic dispensing extrusion-based 3D bioprinter (KIWI 3D printer, Sharebot S.r.l., Italy) (Contessi Negrini et al., 2018). Printed models were designed in SolidWorks, exported as STL files, and finally converted into G-code files (Slic3r software). Ring models (Ø_internal_ = 2 cm, Ø_external_ = 3.6 cm) were printed to assess the ink’s printability (nozzle diameter = 18 G, writing speed = 5 mm/s, *n* = 3). Printed rings were crosslinked in CaCl_2_ 150 mM, and sample height was measured by a digital caliper. The percentage printing accuracy (*Accuracy*_*h*_) (Giuseppe et al., 2018) was calculated by comparing the theoretical height designed by CAD (*h*_*theoretical*_) to the measured height (*h*_*measured*_) of the printed rings (Eq. 1):

$$Accuracy_{h}\left[\%\right]=\left[1-\left|\frac{h_{theoretical}-h_{measured}}{h_{theoretical}}\right|\right]\times 100$$ (1)

Inks printed with accuracy ≥95% were selected as suitable for further investigation; accuracy comprised between 65% and 95% was considered acceptable; inks printed with accuracy ≤65% were not further considered.

### Bioink Selection and Bioprinting

The cytocompatibility of the selected ink formulations (P2.5-C0, P2.5-C0.5, P2.5-C1, and P2.5-C1.5, see Results and Discussion) was investigated by embedding L929 fibroblasts (10 × 10^6^ cells/ml, ECACC No 85011425) in the hydrogel precursors (*n* = 3). After mixing cells with the precursors, hydrogels were crosslinked and cultured in six-multiwell tissue culture polystyrene (TCPS). After 24 h, a live/dead staining (propidium iodide 10 μM and calcein-AM 2 μM) was performed and images (*n* = 6 per sample) were acquired by a fluorescence microscope (Olympus BX51W1). Cell percentage viability was calculated as the ratio of the number of viable cells (i.e., green cells, *N*_*viable*_) to the total number of cells (i.e., green cells + red cells, *N*_*dead*_; Eq. 2):

$$Cell\;viability\;\left[\%\right]=\frac{N_{viable}}{N_{viable}+N_{dead}}\times 100$$ (2)

The P2.5-C1 formulation was selected for bioprinting tests (see Results). The rheological properties of the ink were investigated by thixotropy and temperature sweep tests. Thixotropy tests were performed by applying a constant shear rate (0.1 s^−1^ for 120 s), subsequently increased (100 s^−1^ for 100 s), eventually followed by a recovery phase (0.1 s^−1^, 300 s). Temperature sweep tests were performed by applying a temperature ramp from 10 to 40°C (5°C min^−1^, 1% strain, 1 Hz; the linear viscoelastic region LVR was preliminary checked, data not shown). Printing parameters were then optimized by a serpentine model (Contessi Negrini et al., 2018), composed of six equally long segments (length L = 20 mm), each one alternated with five shorter segments (L = 4 mm). The parameters were optimized by varying the nozzle diameter (18, 20, and 22 G) and writing speed (12, 16, 20, and 24 mm/s). Optimization was performed by considering the printing accuracy (*Accuracy*_*d*_), calculated by comparing the theoretical diameter of the CAD design (840 µm for 18 G, 690 µm for 20 G, 430 µm for 22 G) to the measured diameter (Celestron Micro360 optical microscope) of the printed strands (Eq. 3):

$$Accuracy_{d}\left[\%\right]=\left[1-\left|\frac{D_{theoretical}-D_{measured}}{D_{theoretical}}\right|\right]\times 100$$ (3)

Finally, the printability of 3D structures was investigated by printing four-layer grid structures (20 × 20 mm; pore area = 1.5 × 1.5 mm); each layer was composed by superimposed serpentine pattern models, with 0–90° alternate orientation. The nozzle diameter was set at 20 G; writing speed was varied between 12, 16, 20, and 24 mm/s. The printing accuracy (*Accuracy*_*a*_) was calculated as (Eq. 4):

$$Accuracy_{a}\left[\%\right]=\left[\frac{1}{n}\;\overset{n}{\underset{i=1}{\sum }}\left(1-\frac{\left|A_{i}-A_{t}\right|}{A_{t}}\right)\right]\times 100$$ (4)

where *A*_*t*_ is the theoretical pore area and *A*_*i*_ is the area measured by optical microscope for the evaluated printed pores (number of pores *i* = 45).

*In vitro* tests were conducted on printed and bulk samples, obtained as control by hydrogel casting. Printed structures were designed with disk morphology (Ø = 10 mm, *h* = 2.5 mm), printed by using 5 mm/s writing speed and 20 G nozzle diameter, and crosslinked with CaCl_2_ 150 mM. Stability and percentage weight variation of the printed structures (*n* = 3) were evaluated by swelling tests in DMEM at 37°C, by measuring the weight in time *W*_*t*_ (up to 3 weeks) of the printed and bulk structures and comparing it to their initial weight W_0_ (Eq. 5):

$$Weight\;Variation\;\left[\%\right]=\frac{W_{t}-W_{0}}{W_{0}}\times 100$$ (5)

The solid gel fraction of the hydrogels was calculated after 72 h of swelling, as the ratio of the dry weight of the samples after swelling to the dry weight of the samples before swelling tests (*n* = 3). Mechanical compressive tests (*n* = 3) were performed with a Dynamic Mechanical Analyzer (DMA Q800, TA Instruments). Tests were conducted at 37°C by applying a load/unload compressive cycle up to 30% strain (preload 0.001 N, 2.5% min^−1^ load phase, 5% min^−1^ unload phase) (Michelini et al., 2020).

Finally, bioprinting tests were performed by preparing a bioink composed by the P2.5-C1 formulation and L929 cells (10 × 10^6^ cells/ml). Once printing parameters for complex multilayers structures were optimized by grid printing (see paragraph above), samples were either printed with a three-layers serpentine model (nozzle diameter = 18 G, writing speed = 24 mm/s) or prepared as cell-embedded bulk hydrogels. Cell distribution and viability in the hydrogels were investigated by live/dead staining, as previously described. Cell metabolic activity was measured by alamarBlue® assay (*n* = 3) up to 7 days of *in vitro* culture (Pimenta de Melo et al., 2019).

### Statistical Analysis

Data are expressed as mean ± standard deviation. Data normal distribution was checked by Shapiro–Wilk test. One-way ANOVA tests, unpaired *t*-test, and Mann–Whitney nonparametric test were performed with GraphPad Prism software; *p* < 0.05 was set as statistically significant threshold.

## Results and Discussion

Despite the increasing interest in the use of pectin as biomaterial (Markstedt et al., 2015; Nguyen et al., 2015; Zhao et al., 2016; Neufeld and Bianco-Peled, 2017; Rezvanian et al., 2017; Pereira et al., 2018a; Türkkan et al., 2018; Mehrali et al., 2019; Zhu et al., 2019; Al-arjan et al., 2020; Khotimchenko, 2020; Oh et al., 2020), the low viscosity values of pectin solutions at low concentrations result in poor shape fidelity after 3D bioprinting, thus limiting the use of pectin as bioink (Methacanon et al., 2014). A potential solution is represented by the preparation of multicomponent inks, in which pectin and other polymer(s) can be mixed and crosslinked to obtain a multicomponent hydrogel to increase pectin printability while simultaneously maintaining its properties (Cui et al., 2020).

Here, we designed and prepared multicomponent bioinks based on pectin and TOCNFs (P-C) to obtain printable pectin-based bioinks. TOCNFs were selected as additional component thanks to their ability in enhancing the ink’s high viscosity and shear-thinning behavior, which improved both printability and shape fidelity after printing (Martínez Ávila et al., 2016; Cui et al., 2020; Fiorati et al., 2020). Moreover, TOCNFs demonstrate adequate biocompatibility (Cui et al., 2020; Fiorati et al., 2020) and can be used as main component of bioinks or as reinforcement of multicomponent bioinks (Cernencu et al., 2019). We first performed preliminary 3D printing studies by preparing P-C multicomponent hydrogels in distilled water. We combined different concentrations of pectin (0%, 1%, and 3% w/v) and TOCNFs (0%, 0.5%, 1%, 2%, and 3% w/v) to print ring structures (Supplementary Figure S2A). Enhanced printing accuracy was achieved by increasing either pectin or TOCNFs concentrations (Supplementary Figure S2B). Addition of TOCNFs was necessary to print self-standing cylinders that did not collapse during the layer-by-layer additive process. However, despite good printing accuracy being achieved, the prepared hydrogels showed acidic pH of approximatively 3.5 (Supplementary Figure S3A) (Moreira et al., 2013). The acidic pH and the use of water to prepare the inks heavily affected the viability of L929 cells encapsulated in the hydrogels (Supplementary Figure S3B). We then subsequently prepared our inks in DMEM and adjusted their pH at 6 to 7 to guarantee a cytocompatible hydrogel (Supplementary Figure S3B).

**FIGURE 1:**
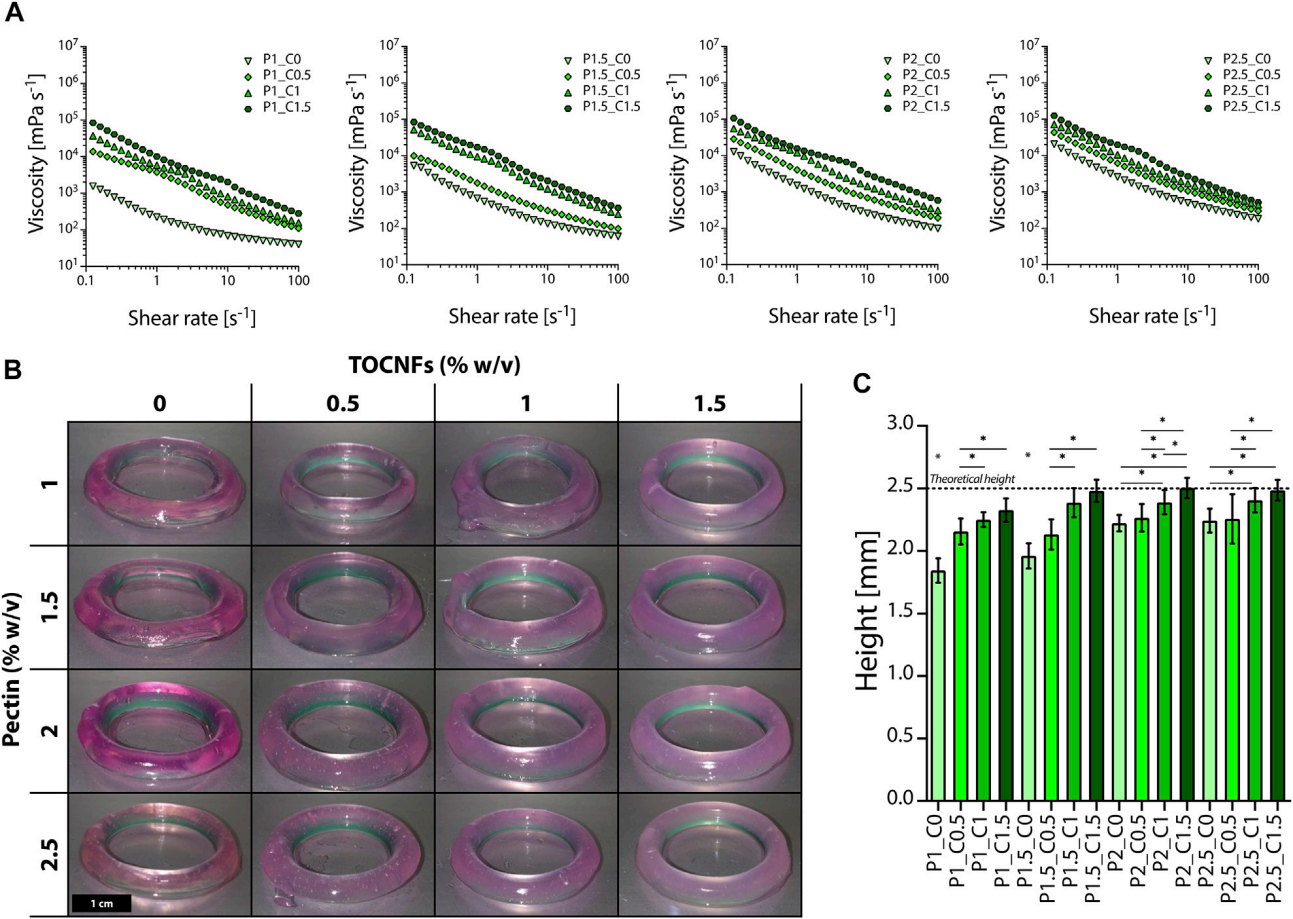
Evaluation of the printability of pectin-TOCNFs inks. **(A)** Shear rate ramp rheological tests on inks prepared by varying the concentration of pectin (P1, P1.5, P2, and P2.5) and TOCNFs (C0, C0.5, C1, and C1.5). **(B)** Macroscopic images of rings printed by varying the multicomponent inks composition (scale bar = 1 cm). **(C)** Comparison between the measured height of the printed rings and the theoretical height (dot line); *p < 0.05.

Despite the first tests allowing us to improve the printability of the inks prepared in water, low accuracy was achieved when relatively low concentrations were used. Moreover, correction of the acidic pH to improve cell viability critically diminished viscosity and affected the hydrogels’ printability (Moreira et al., 2013). Thus, we partially pre-crosslinked the inks by addition of CaCl_2_ to increase their viscosity and obtain printable inks (Yu et al., 2016; Vancauwenberghe et al., 2017, 2018). All the inks showed shear-thinning response under shear rate ramps (Figure 1A), with viscosity values decreasing by increasing the applied shear rate (Methacanon et al., 2014; Wang et al., 2014). This shear thinning rheological response has to be attributed to the pectin polysaccharide structure, where the reduction in viscosity is given by polymer chains alignment of transiently elongated coils in the flow direction during the application of a shear rate ramp as previously described for pectin and other polysaccharides (Zhang et al., 2013; Wang et al., 2014; Campiglio et al., 2020). Higher viscosity values were observed both by increasing the pectin and TOCNFs concentration. When the same TOCNFs concentration is considered, increased viscosity characterized hydrogels prepared with higher concentration of pectin, due to the higher number of hydrogen bonds formed thanks to the increased density of hydroxyl groups forcing the mutual interpenetration between pectin polymer chains. Interpenetrated pectin molecules limit hydrogel flowing and viscosity solution increases (Methacanon et al., 2014; Colodel et al., 2019). This phenomenon can be particularly observed when low TOCNFs concentrations are considered (0, 0.5% w/v). For higher TOCNFs concentrations (1%, 1.5% w/v), the relatively high viscosity achieved by addition of TOCNFs partially masked the contribution of pectin to the rheological properties of the inks. Moreover, for all the tested pectin concentrations (Table 1), addition of TOCNFs led to an increase of viscosity of the inks, promising for the development of printable multicomponent bioinks (Markstedt et al., 2015; Sawkins et al., 2015; Henriksson et al., 2017; Müller et al., 2017; Nguyen et al., 2017; Cui et al., 2020; Piras and Smith, 2020). Viscosity increased by the addition of TOCNFs, while the shear thinning properties of the inks were maintained, given that nanocellulose-based inks exhibit a high zero-shear viscosity and shear-thinning behavior even at low polymer concentration (Martínez Ávila et al., 2016). Moreover, previous 3D printing studies on nanocellulose-based hydrogels showed that nanocellulose is a promising ink additive that effectively improves not only mechanical properties and flowability, but also enhances chemical and biocompatible properties of bioinks (Xu et al., 2018).

**FIGURE 2:**
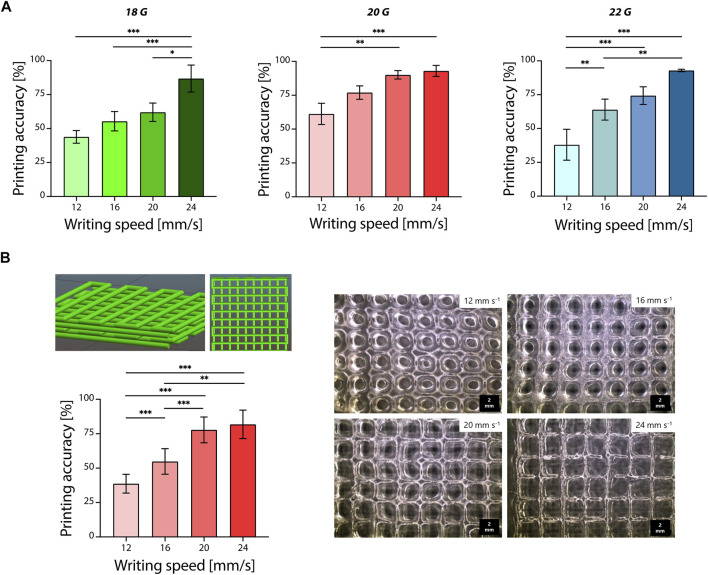
Optimization of pectin-TOCNFs inks 3D printing. **(A)** Printing accuracy evaluated by varying the nozzle size (18, 20, and 22 G) and writing speed (12, 16, 20, and 24 mm/s); *p < 0.05; **p < 0.01; ***p < 0.001. **(B)** Optimization of the printing of 3D grid structures by varying the writing speed (12, 16, 20, and 24 mm/s, **left**) and microscopy images of the obtained 3D printed structures (scale bar = 2 mm, **right**); **p < 0.01; ***p < 0.001.

We then investigated the biomaterial printability to narrow the set of inks to be tested for printing parameters optimization and bioprinting, by evaluating the height of the printed cylinders (Figure 1B) and the capability of self-sustaining their structure during the layer-by-layer additive procedure (Campos et al., 2015; Contessi Negrini et al., 2018; Giuseppe et al., 2018). All the prepared inks were successfully extruded during the printing process and printed in the desired shape (Figure 1B). Considering fixed pectin concentrations, an increase in the height of the printed rings was observed by increasing the TOCNFs concentration, thus confirming TOCNFs influence in increasing the inks’ viscosity and their printability (Figure 1C). Furthermore, pectin concentration also influenced the inks’ printability since the height of the cylinders increased with increasing pectin concentration. Similar trends were observed in studies involving nanocellulose as reinforcement in different polymer-based composite inks, bringing benefits to rheological and mechanical properties of printed composite materials (Cernencu et al., 2019). Addition of nanocellulose to alginate-based bioinks led to an improved geometrical resolution and a high shape fidelity, due to the obtained highly viscous, shear-thinning multicomponent bioink (Martínez Ávila et al., 2016). Similarly, a macroscopic loss in shape fidelity was observed by decreasing nanocellulose concentration in alginate-based bioinks (Heggset et al., 2019). In our work, TOCNFs critically contributed to enhancing the inks’ viscosity and allowed printing structures with heights comparable to the theoretical ones designed by CAD (Figure 2C). Low TOCNFs concentration gels exhibited height values far from theoretical value, showing a low printing accuracy (≤65%). As the highest printing accuracy was achieved by printing inks with 2.5% pectin concentration (≥95%), this set of inks (P2.5-C1.5, P2.5-C1, P2-C1.5, and P1.5-C1.5) was selected to further develop the multicomponent bioinks.

After fixing the pectin concentration (2.5% w/v), we investigated the influence of the TOCNFs concentration on the viability of cells encapsulated in the hydrogels. For all the considered hydrogel formulations, percentage cell viability was higher than 80% (Supplementary Figure S4). However, a decrease in cell viability was observed for the highest TOCNFs concentration tested (P2.5-C1.5, *p* < 0.05 vs. the other formulations). The P2.5-C1 ink formulation was selected for printing parameters optimization and bioprinting tests, due to the good compromise between satisfactory printing accuracy and adequate cell viability.

The selected P2.5-C1 ink formulation showed a good viscosity recovery during thixotropy rheological tests (Supplementary Figure S5A, left). This formulation showed a constant viscosity in the initial phase (low shear rate) and in the subsequent intermediate region. Subsequently, when the shear rate was decreased, the hydrogel completely recovered its initial viscosity values (100%) in 10 s. No significant variation in the rheological properties of the inks were detected by varying the testing temperature (Supplementary Figure S5B, right) and the storage modulus G’ was predominant on the loss modulus G”.

Optimization of the printing parameters was then performed by varying the nozzle size and the writing speed used to print the P2.5-C1 ink formulation (Figure 2). Printing accuracy was evaluated by measuring the diameter of the printed filaments and by comparing it to the nozzle size. Printing accuracy was relatively low (<70%) for writing speeds equal to 12, 16, and 20 mm/s, but it was improved (accuracy >75%) when writing speed was increased for all the considered nozzles (Figure 2A). The highest values of printing accuracy were obtained by printing at the maximum tested writing speed (24 mm/s) and were equal to 86% ± 10%, 93% ± 4%, and 93% ± 1% for 18, 20, and 22 G, respectively. The achieved accuracy led to a better replication of the CAD-designed 3D models, since printed ink could be extruded in defined strands on the printing platform, avoiding excess of material accumulation during the printing process, as described for other natural-based polymers printed by extrusion-based technology (Contessi Negrini et al., 2020; Tian et al., 2021; Zhao et al., 2021).

Once printing parameters were optimized on serpentine pattern models, four-layer complex grid structures were investigated for 3D printing (nozzle diameter = 20 G, writing speed = 12, 16, 20, and 24 mm/s). For this purpose, 20 G nozzle diameter was selected to ensure accurate material deposition and optimal printing accuracy (>75%). Printing accuracy was evaluated by considering the pore area, a fundamental parameter to obtain porous scaffolds for tissue engineering and biomedical applications (Zheng et al., 2020). As for the previous 2D optimization, the accuracy of the printed scaffolds in terms of pore geometry was increased by increasing the printing writing speed (Figure 2B, left). The printing accuracy was qualitatively confirmed by the observed morphology of the printed scaffolds (Figure 2B, right). Pores became morphologically well-defined at higher writing speeds (20 and 24 mm/s), and the calculated printing accuracy was acceptable (>75%). Lower writing speeds (12 and 16 mm/s) led to an undesired accumulation of printed material resulting in rounded morphology pores. Consequently, pore area decreased due to filaments spread and merging on each other (Giuseppe et al., 2018). These printing parameters were then selected to test the potential use of the optimized ink formulation (P2.5-C1) as bioink.

**FIGURE 3:**
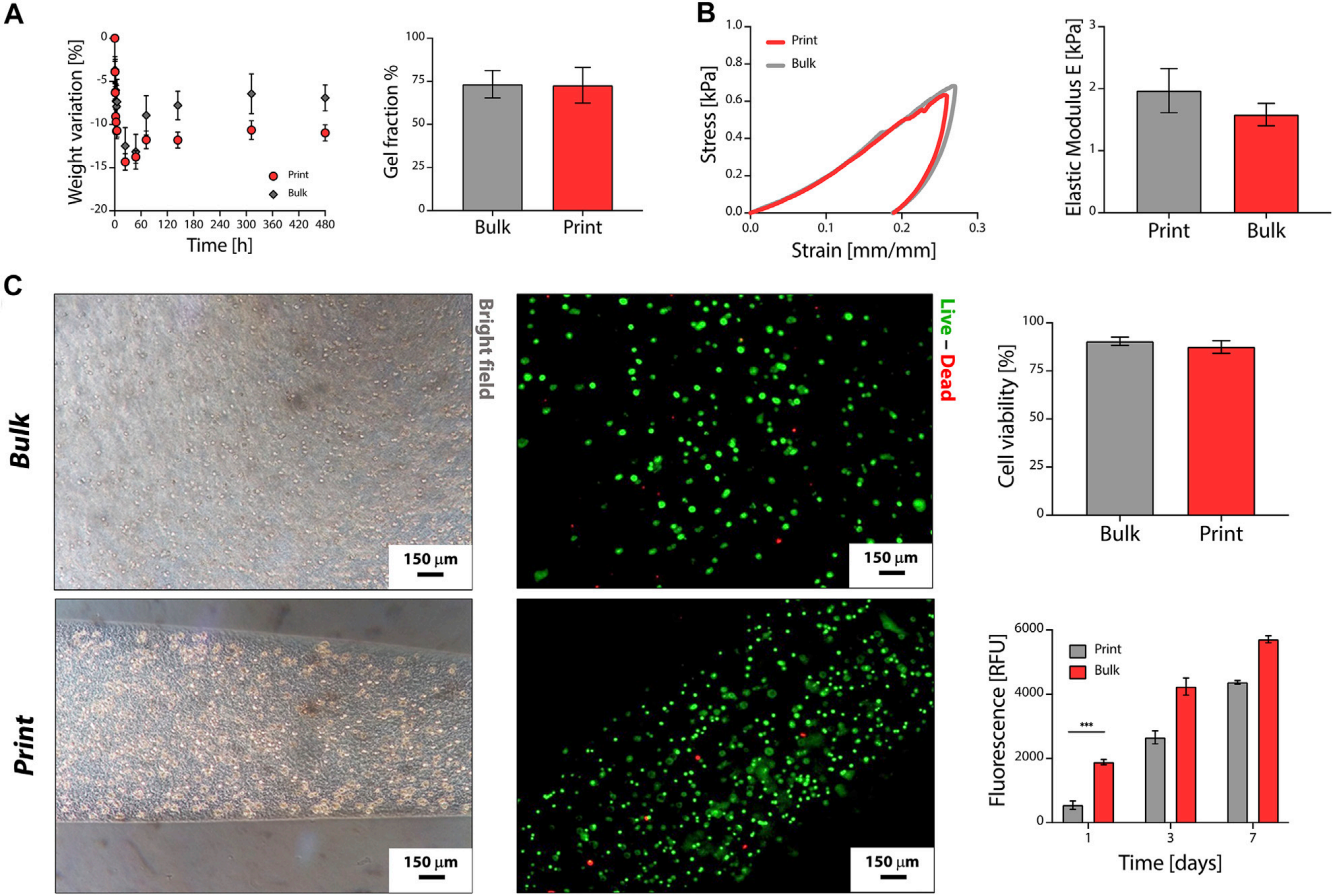
Pectin-TOCNFs bioprinting. **(A)** Weight variation **(left)** and solid gel fraction **(right)** of the printed and bulk multicomponent hydrogels. **(B)** Compressive mechanical properties. Representative stress–strain curves **(left)** and elastic modulus E **(right)** of printed and bulk hydrogels. **(C)** *In vitro* cytocompatibility tests and bioprinting. Live/dead staining of cells embedded in bulk and printed hydrogels **(left)** and percentage cell viability **(top right)**. Metabolic activity, tested by alamarBlue® assay, of cells cultured in bulk and printed hydrogels **(bottom right)**; ***p < 0.001.

*In vitro* tests were performed to compare the performance of P2.5-C1 print and bulk hydrogels. The printed ink was stable after immersion in physiological-like conditions (Figure 3A, left). Both print and bulk samples were characterized by an initial weight loss (approximatively 25% of solid gel fraction) that can be attributed to dissolution of non-crosslinked polymer chains. The loss of solid fraction was confirmed by gel fraction tests (Figure 3A, right), which revealed a solid gel fraction of both print and bulk hydrogels of approximatively 75% after 72 h of swelling (*p* > 0.05). After 72 h of immersion, sample weight was stable for up to 3 weeks, proving the successful obtainment of crosslinked hydrogel networks stable in physiological-like conditions. Weight variation at plateau was higher for print samples compared to bulk hydrogels. The bulk and printed hydrogel showed typical viscoelastic behavior when compressed (Figure 3B, left). The elastic moduli of bulk and print samples were comparable (*p* > 0.05) and in the range of soft hydrogels (2.0 ± 0.4 kPa and 1.8 ± 0.2 kPa for printed and bulk samples, respectively; Figure 3B, right).

Finally, the potential use of the developed multicomponent ink biomaterials as bioinks was assessed (Figure 3C). Cells were evenly homogeneously distributed 3D in the hydrogels both considering bulk and print samples (Figure 3C, left). Viable cells (green cells in Figure 3C) were observed in all the samples, showing the presence of viable cells distributed in the hydrogels, even after the printing process. The percentage cell viability was higher than 85% for both print and bulk formulations (*p* > 0.05, Figure 3B, top right). After 1 day of culture, higher metabolic activity was observed for bulk samples (*p* < 0.05; Figure 3C, bottom right), possibly due to the shear rate exercised on cells by the extrusion process. Cell metabolic activity increased during the *in vitro* culture for both print and bulk samples (*p* > 0.05) up to 7 days of culture.

## Conclusion

Multicomponent inks obtained using pectin and TOCNFs were designed and investigated in this work. Optimization of the ink formulation was achieved by a compromise between the printing accuracy and cell viability in the developed hydrogels. Geometries reproducing the CAD design were successfully printed. The printed scaffolds were stable in physiological-like environment, showing the successful obtainment of crosslinked pectin-TOCNFs hydrogels. Viable cells were printed using the optimized ink formulation and printing parameters, showing the potential use of the developed biomaterial inks as bioinks for future biofabrication applications.

## Data Availability

The raw data supporting the conclusion of this article will be made available by the authors, without undue reservation.
