# Supplementary material for: 3D Bioprinting of Pectin-Cellulose Nanofibers Multicomponent Bioinks
Source: Front Bioeng Biotechnol. 2021 Dec 3;9:732689. doi: 10.3389/fbioe.2021.732689 (PMC8678092; doi:10.3389/fbioe.2021.732689)
Supplement: Supplementary file 1 [file DataSheet1.PDF]

3D bioprinting of pectin-cellulose nanofibers multicomponent bioinks

Supplementary Material

Supplementary Material 1. Synthesis of TOCNFs. TEMPO-Oxidized Cellulose NanoFibers (TOCNF) were synthetized by using the following procedure. 2,2,6,6-tetramethylpiperidine-1-oxyl radical TEMPO (538 mg, 3.44 mmol) and potassium bromide (3.70 g, 31.1 mmol) were dissolved in 0.7 L of deionized water. Separately, cellulose from cotton linters (25 g) was dispersed in deionized water (0.7 L) and then added to the solution containing TEMPO and KBr. Sodium hypochlorite (12.5% w/w aqueous solution, 155 mL) was gradually added to the reaction slurry in 2 h, under constant agitation. The pH of the reaction was continuously adjusted to values in the range 10.0-10.5 by dropwise addition of NaOH 4 M. The reaction mixture was then left to react for 16 h and eventually acidified to pH 2 by addition of HCl 37% w/w. The white solid was collected by filtration and washed with deionized water (5×250 mL) and ethanol (2×100 mL). Once dried, TEMPO-oxidized cellulose was obtained (22.5 g, 90% yield). The number of carboxylic groups was colorimetric titrated with NaOH and phenolphthalein (1.76 $mmol_{COOH}/g_{cellulose}$).

TOCNFs stock dispersions were prepared by adding to the desired amount of TEMPO oxidized cellulose 1 equivalent of $NaOH_{aq}$ 0.1 M (with respect to the carboxylic groups content), and the volume was adjusted with water (e.g. to prepare 3% w/w TOCNFs dispersion: 0.3 g of TEMPO oxidized cellulose, 5.28 mL of $NaOH_{aq}$ was added, and the final volume was adjusted to 10 mL with water). The mixtures were then sonicated (Branson Sonifier 250, 6.5 mm probe tip) until clear solutions were achieved (about 20 min, 0 °C). The pH of the TOCNFs stock dispersion was adjusted to 7.0 by addition of $NaOH_{aq}$ 0.01 M or HCl_{aq} 0.01 M.

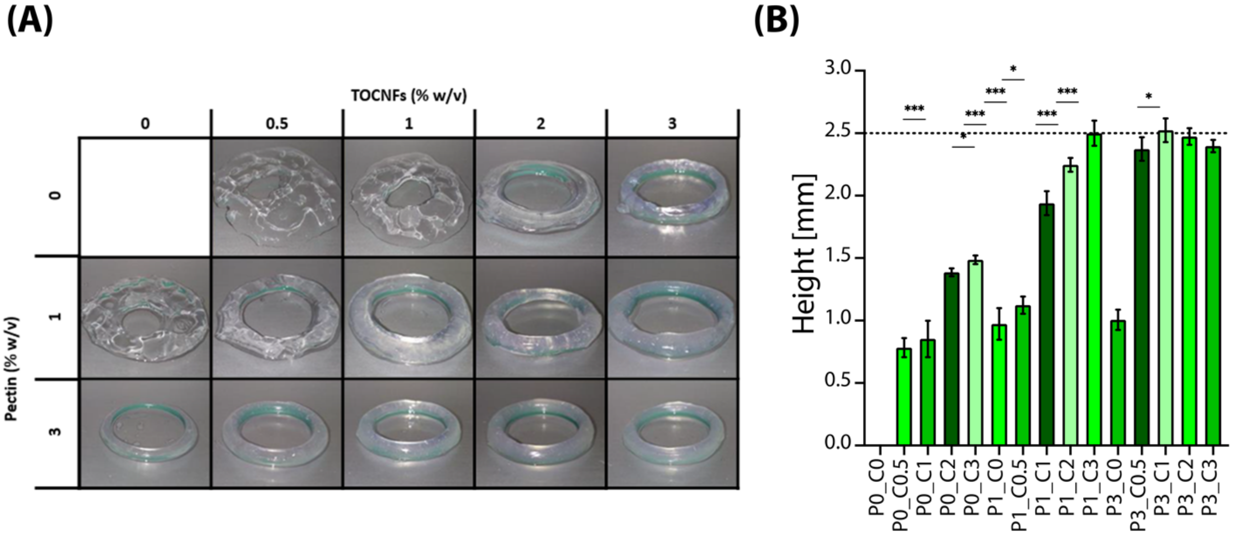

Supplementary Material 2. Preliminary printability tests of pectin (0, 1, and 3% w/v) and TOCNFs (0, 0.5, 1, 2, and 3% w/v) multicomponent biomaterial inks prepared in distilled water. **(A)** Ring structures ($Ø_{int}$ = 2 cm, $Ø_{ext}$ = 3.6 cm) were printed using an 18 G nozzle and 5 mm/s writing speed. At completed printing, hydrogels were crosslinked using $CaCl_2$ 150 mM for 6 minutes. The cylinders height was then measured in 6 different points of the structures and **(B)** the printing accuracy was calculated following Equation 1 (main text); * $p < 0.05$, *** $p < 0.001$.

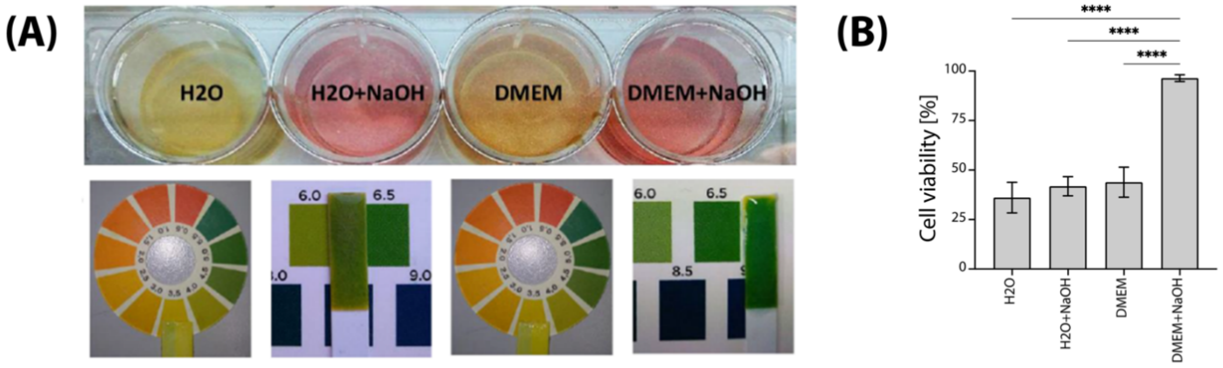

Supplementary Material 3. Optimization of pH for bioinks development. **(A)** pH was measured for P-C aqueous solutions, P-C aqueous solutions after pH adjustment via NaOH, P-C solutions in DMEM, P-C solutions in DMEM after pH adjustment via NaOH. *In vitro* evaluation of viability of cells encapsulated in the hydrogels. **(B)** Percentage cells viability measured by live/dead staining 24 h after the encapsulation of L929 cells in pectin hydrogels; **** $p < 0.0001$.

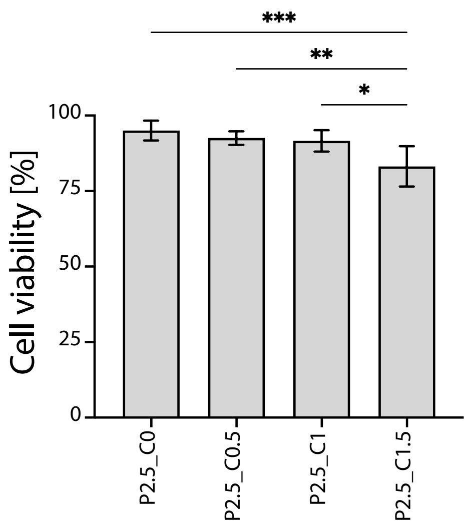

Supplementary Material 4. Percentage viability of L929 fibroblast cells embedded in selected pectin and TOCNFs multicomponent ink combinations (i.e., P2.5-C1.5, P2.5-C1, P2-C1.5, P1.5-C1.5); * $p < 0.05$, ** $p < 0.01$, *** $p < 0.001$.

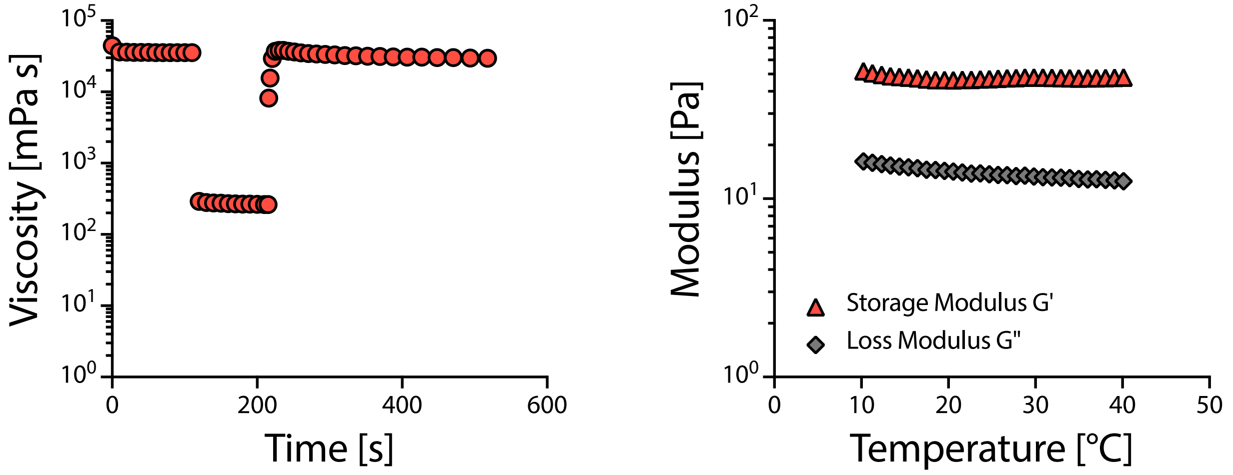

Supplementary Material 5. Rheological characterization of the selected ink formulation (P2.5-C1). **(A)** Thixotropy tests. Tests were performed by applying a constant shear rate, subsequently increased, and the hydrogel was allowed to recover in the last part of the test. **(B)** Temperature sweep tests. Storage modulus (G') and loss modulus (G") evaluated by increasing the temperature**.**
